# The Potential of Remedial Techniques for Hazard Reduction of Steel Process by Products: Impact on Steel Processing, Waste Management, the Environment and Risk to Human Health

**DOI:** 10.3390/ijerph16122093

**Published:** 2019-06-13

**Authors:** Kiri Rodgers, Iain McLellan, Simon Cuthbert, Victoria Masaguer Torres, Andrew Hursthouse

**Affiliations:** 1 School of Health and Life Science, University of the West of Scotland, Paisley PA1 2BE, UK; kiri.rodgers@uws.ac.uk; 2 Engineering and Physical Sciences, School of Computing, University of the West of Scotland, Paisley PA1 2BE, UK; Iain.mclellan@uws.ac.uk (I.M.); Simon.cuthbert@uws.ac.uk (S.C.); 3 ArcelorMittal Global R&D Asturias, Marques de Suances s/n - Apartado 90, 33400 Avilés, Spain; victoria.masaguer@arcelormittal.com; 4 Hunan Regional Key Laboratory for Shale Gas Resource Exploitation, Hunan University of Science and Technology, Xiangtan 411201, China

**Keywords:** waste cleaning technologies, steel process by-products, remediation of hazardous waste

## Abstract

The negative impact from industrial pollution of the environment is still a global occurrence, and as a consequence legislation and subsequent regulation is becoming increasingly stringent in response, in particular, to minimising potential impact on human health. These changes have generated growing pressures for the steel industry to innovate to meet new regulations driving a change to the approach to waste management across the industrial landscape, with increasing focus on the principles of a circular economy. With a knowledge of the compositional profiles of process by-products, we have assessed chemical cleaning to improve environmental performance and minimise disruption to manufacturing processes, demonstrating re-use and recycling capacity. We show that with a knowledge of phase composition, we are able to apply stabilisation methods that can either utilise waste streams directly or allow manipulation, making them suitable for re-use and/or inert disposal. We studied blast furnace slags and Portland cement mixes (50%/50% and 30%/70%) with a variety of other plant wastes (electrostatic precipitator dusts (ESP), blast furnace (BF) sludge and basic oxygen furnace (BOF) sludge) which resulted in up to 90% immobilisation of hazardous constituents. The addition of organic additives i.e., citric acid can liberate or immobilise problematic constituents; in the case of K, both outcomes occurred depending on the waste type; ESP dust BF sludge and BOF fine sludge. Pb and Zn however were liberated with a 50–80% and 50–60% residue reduction respectively, which generates possibilities for alternative uses of materials to reduce environmental and human health impact.

## 1. Introduction

In 2017 approximately 1.69 billion tonnes of crude steel was produced worldwide [1] with as much as 400 kg of solid waste being generated per tonne of steel and requiring disposal [2]. In the last three decades a number of methods for the recovery and reuse of waste by-products (powdered wastes, flue dusts, slag, and sludge) have been developed for application within the steel manufacture industrial life cycle, however, as a result of the pressure from continuous reduction in regulatory classification thresholds worldwide, standard cleaning operations are struggling to meet the legal requirements [3,4]. Thresholds from compliance leaching tests across Europe: EN 12457 [5], and UK BS EN 12457 [6] known as Waste Acceptance Criteria (WAC) testing has forced the need for investment in alterations to and/or additional steps in the steel production process [7,8]. The major contaminants of concern are cadmium (Cd), copper (Cu), chromium (Cr), lead (Pb), nickel (Ni), potassium (K) and zinc [9,10,11,12]. This research focuses on K, Pb and Zn.

**Figure 1:**
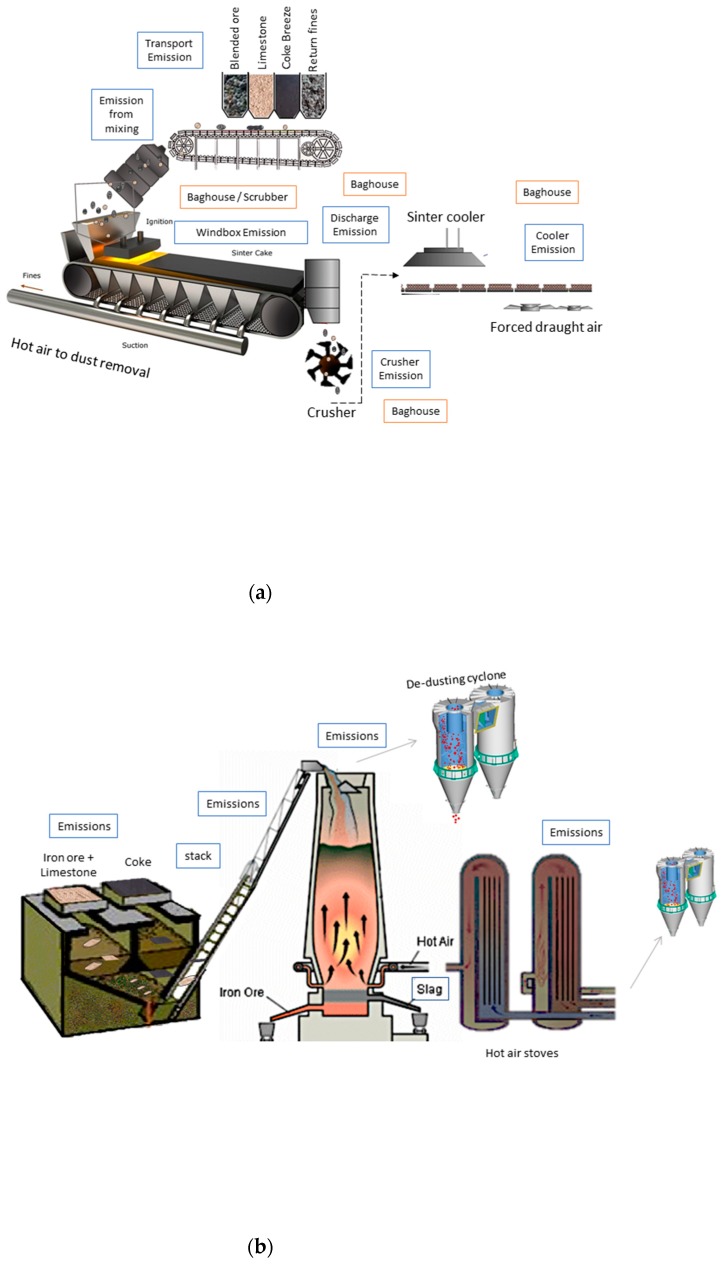

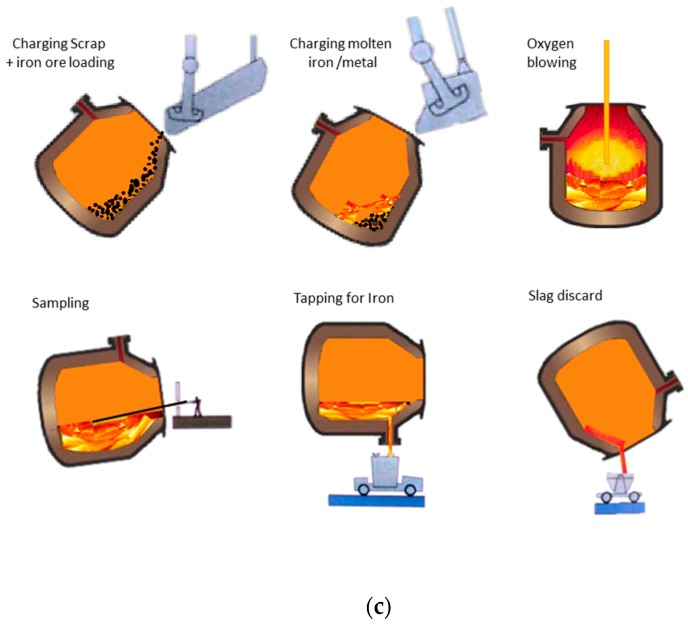
Schematic diagram of steel production highlighting process by-product emissions points in typical steel plant operations: (**a**) Sinter plant; (**b**) blast furnace and (**c**) basic oxygen furnace (a generic summary based on industry process descriptions and site visits to a number of manufacturing plants).

The modern production of steel is a complex process even when processing is simplified. It includes a diverse range of emission and abatement systems (Figure 1), and although cleaning treatments have previously been applied [13,14,15,16,17,18,19,20,21,22,23,24,25,26,27], there is still a lack of information on their impact on waste processing. Established treatment techniques are based on pyrometallurgy, hydrometallurgy or a combination of both methods, with the latter offering processes favoured as a treatment which offers the best economic return with a higher degree recovery for valuable metals [28,29]. However, the processes have recognised limitations and struggle to digest zinc ferrites, which is dominant form of zinc in baghouse dusts [30]. Zinc is both of value as a process alloying material e.g., brass [31], as a by-product recycled back into sinter [32,33], but can also have a deleterious effect on steel production if built up through excessive use of recycled metals e.g., from a process feed of galvanised product wastes, or in the feed itself. Furthermore, it can cause structural damage to the plants themselves [34,35,36]. Since emission controls can only be applied by altering the production process or removing the cause during production, coupled with the 365-day operational approach for competitive steel manufacturing, the options for emission reduction are through suppression, extraction, and/or abatement [37].

With these restrictions the ability to treat industrial wastes by immobilisation or removal of hazardous components in order to alter their disposal classification i.e., hazardous, non-hazardous, inert, can offer great potential to reduce industrial costs and address environmental impact. Immobilisation can take place by applying techniques such as solidification or stabilization (S/S) that use binding reagents to immobilize the hazardous constituents. The approach for S/S can often be coupled together as one can occur as a result of the other which and the reason for S/S being frequently identified together. These approaches are widely used for treatment of hazardous wastes that are mostly inorganic (aqueous wastes, sludge, slags, dust, and ashes containing hazardous metals) and contaminated soils before final disposal [38].

By utilising experience from previous studies of solidification/stabilisation (S/S) [9,39,40,41] using binding/bulking agents [9,42,43,44,45,46,47,48,49], or organic additives [50,51,52,53,54,55,56,57,58,59] we can evaluate the potential of alternative processes to develop more sustainable production strategies. Thus focusing on reducing production costs and impacts on direct negative environmental and human health risk whilst increasing production efficiency, resource utilisation and a reduction in the amount of waste generated [22]. This will not only reduce emission thresholds to meet legal requirements, but also help minimise any future risk to human and wider environmental impact these contaminants have [60].

This research focuses on a number of elemental groups: key potentially toxic elements (PTEs) of interest (K, Pb, Zn), associated elements (Al, Ca, Fe and Mn) and additional elements of interest (Cr, Cu, Mn and Ni). The associated elements Al, Ca, Fe and Mn are elements that can have a strong influence upon the liberation of the priority constituents of interest (K, Pb and Zn). The Al and Si content dictates the acidity or buffering capacity of solid samples. Here only Al is reported because Si could not be measured due to instrumental limitations. The elements Ca, Mn and Fe are identified because the acidic conditions created by the citrate solution can lead to the dissolution of iron/manganese hydroxides, which releases through desorption of the associated PTEs [50]. Additional elements Cr, Cu, Mn and Ni are often reported as components of steel waste and are known problematic PTEs environmentally [10,11,61,62].

Furthermore, citrate can be highly effective in enhancing the solubilisation of sparingly soluble inorganic iron hydroxides (Fe) in soils. Interestingly, this creates an enhanced sorption system for alternative and potentially more strongly complexed metallic components consequently enhancing their [51]. The calcium ion binds in one of two ways; a single citrate species can bind one calcium ion or two citrate molecules to one calcium ion causing a collision-induced dissociation of precursor ions, metal:citrate stoichiometry is favoured [63].

We report here on an assessment of the potential benefits from treatment strategies for steel wastes through reduction of hazard potential and the implications for wider management processes in the steel life cycle.

## 2. Materials and Methods

### 2.1. Analytical Procedures and Quality Assurance/Quality Control

Samples were supplied from steel plants, air-dried, sieved to less than 2 mm, mixed, cone and quartered () before being weighed out for experimentation. A series of samples from active plant operations were collected by site operatives. A total of nine Electrostatic Precipitator (ESP) samples were supplied from a sinter plant (see Figure 1a); four blast furnace (BF) air pollution control sludge samples (Figure 1b) and a typical Basic Oxygen Furnace (BOF) fine sludge. All experimental details, reagents, reference materials and instrumental analysis (ICPAES) are detailed in our previous report [64] which includes details of sample and experimental integrity and appropriate levels of quality assurance.

### 2.2. Stabilisation of Steel Making Dust and Sludge

The stabilisation of steel wastes (sludge or dusts) was carried out using S/S techniques, with either the addition of blast furnace (BF) slags or treatment with citric acid washes. Both underwent leaching tests with water and total concentrations are compared to crude waste by-product leachates for treatment evaluations.

#### 2.2.1. Stabilisation by Organic Additive: Leaching with Citric Acid

This kinetic sorption/desorption experiment initially underwent method development [37] to optimise waste/reagent contact and wider experimental protocol and is not discussed in detail here. However, in summary, leaching solutions were prepared from trisodium citrate (Analytical purity, Thermo Fischer) in ultra-high purity water (UHP, 18 Mohm-cm, TripleRed, Bucks). Extraction was performed in 50 mL polypropylene centrifuge tubes with 1 ± 0.1 g samples and 20 mL leaching solution. Shaken for an optimised 44 ± 2 h period on a rotary shaker (Stuart Rotator SB3) before centrifuging 3800 rpm, filtering prior to analysis by ICPAES, with appropriate dilution, as described previously [64]. Replicates were performed for each extraction.

For this study, the effect of citrate solutions at 0.1 and 0.05 M concentrations were compared over different time periods (6, 18, 24, 36 and 48 h) to determine if and when equilibrium was met for optimum treatment conditions and carried out in duplicate. As with adsorption/sorption experiments the interface between the mineral and solution times can affect the way metal ions complex or exchange on surface sites i.e., longer reaction times are expected to generate more stable inner-sphere complexes [65]. This led to the identification of a treatment using a 0.1 M citrate solution shaken for 44 h ±2. This time period highlights the slow equilibrium process and implications for application of treatment within the industrial cycle.

The elements analysed and evaluated, where detected, were: Al, Ca Cr, Cu, Fe, Mg, Mn, Pb and Zn. Sample replicates were good with a median RSD for the majority of PTEs < 10% however Al, Mg and Ni showed higher RSDs of 11.8%, 14.0% and 25.2% respectively.

#### 2.2.2. Stabilisation with Blast Furnace Slag

Steel process wastes (sludge or dust) were mixed with blast furnace slag (BF) with a series of different *w*/*w* ratios; 50:50, 30:70 and 70:30, with minimal water added to generate a stiff paste. The mixing was performed in acid washed beakers using a mechanical whisk (also acid washed). Dry materials were mixed alone for 5 min before adding de-ionised water, typically of 0.4 *v*/*w* ratio or less where possible and mixed for a further 10 min. Samples were stored in acid washed disposable beakers and cured for a minimum of 28 days. Samples were then crushed to pass through a 4 mm sieve in order to put through the WAC BS EN1245-7 leaching protocol. A series of WAC tests were commissioned from an independent external accredited testing laboratory to ensure industry standard performance monitoring and calibrate effect of treatment methods.

## 3. Results

### 3.1. Application of Citric Acid

All sample types are presented as median values and comparisons are made of the treated leachate (mg/kg) to concentrations from Waste acceptance criteria (WAC) leachates. Each graph has been separated into its corresponding categories: sinter ESP dust (samples 1, 2, 3, 4, 5), blast furnace sludge (6, 7) and BOF fine sludge (8).

All data points are quoted as mg/kg and due to ranges of concentration some results have been displayed using a logarithmic scale in order to emphasise trends rather than the variation in elemental concentrations.

#### 3.1.1. Key Elements of Interest

**Figure 2:**
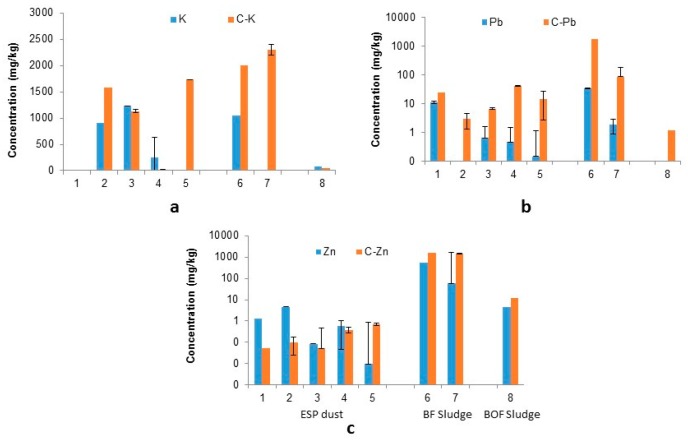
Concentration of (**a**) K, (**b**) Pb and (**c**) Zn (mg/kg) in solution after water and citric acid leach (C-Element refers to citrate-leached results) (Samples ESP dust: 1–5, BF sludge: 6–7, BOF sludge: 8).

Concentrations for K, Pb and Zn can be seen below (Figure 2) highlighting the different concentrations leached by water (WAC) and by 0.1 M citrate solution.

The key constituents Pb and Zn show a similar pattern for all sample types (ESP dust, BF sludge and BOF fine sludge). As expected, a noticeable increase in their release (Table 1) was observed after sample exposure to citrate solution compared to water. Lead shows the highest percentile increase for all sample types (113–1.08 × 10^5^%). Zinc also follows the same trend (59–2382% increase) with the exclusion of ESP dust with a reduction in leached concentration (0–97% decrease). This is indicative of immobilisation, or a result of low measurable levels, which are close to the limit of detection.

Potassium follows this trend of liberation only for sinter ESP dust (excluding sample 3 which yielded an 8% reduction in leached content relative to water) and BF sludge, (74.4–1.73 × 10^8^%, and 90–2.3 × 10^8^% increase respectively).

#### 3.1.2. Associated Elements (Al, Ca, Fe and Mn)

**Figure 3:**
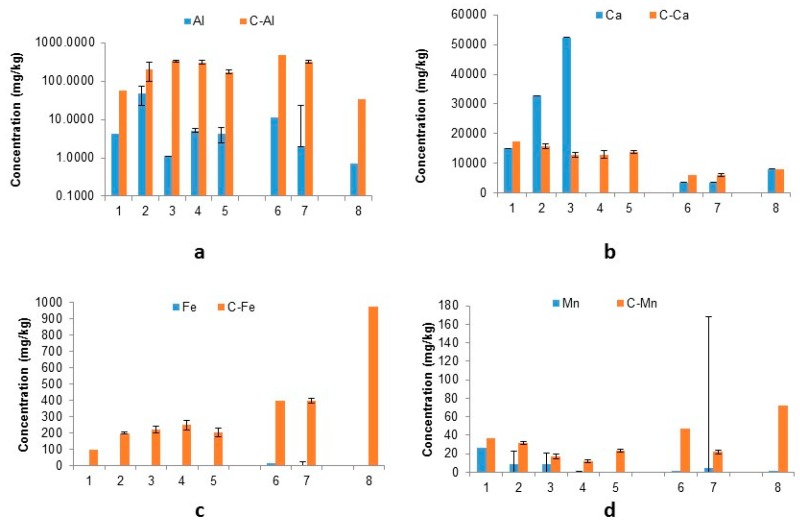
Concentration of: (**a**) Al, (**b**) Ca, (**c**) Fe and (**d**) Mn (mg/kg) in solution after water and citric acid leach (C-Element refers to citrate-leached results) (Samples: ESP dust: 1–5, BF sludge: 6–7, BOF sludge: 8).

The competition between citrate molecules and contributing elements for sorption sites can be influenced by parameters such as individual stability constants and therefore influence the dissolution of key elements of interest (i.e., K, Pb, Zn). The resulting leachate concentrations pre and post treatment can be seen below (Figure 3).

It can be seen that the amount of aluminium leached (mg/kg) significantly increased with the treatment of citrate solution for all sample types with high percentage increases also observed (328–29,369%; Table 2). This trend of liberation can also be seen with iron (2745–2.49 × 10^8^% increase) and Mn (40–3.36 × 10^5^%).

Calcium, however, showed a more unpredictable trend where the ESP dusts (with the exception of samples 4 and 5) showed an immobilisation of Ca after citrate treatment with lower concentrations leached out (52–75% decrease). This is indicative of an insoluble calcium citrate salt potentially precipitating out of solution upon cooling. Sample 1 showed a slight increase in the amount of calcium leached by citrate (15% greater than water) making these observations inconclusive at this stage. The BF sludge samples both showed an increase in leached Ca after treatment with citrate solution as opposed to water (65–77%) and BOF fine sludge shows a negligible relative decrease in concentration (3%).

#### 3.1.3. Additional Elements of Interest (Cr, Cu, Mg and Ni)

**Figure 4:**
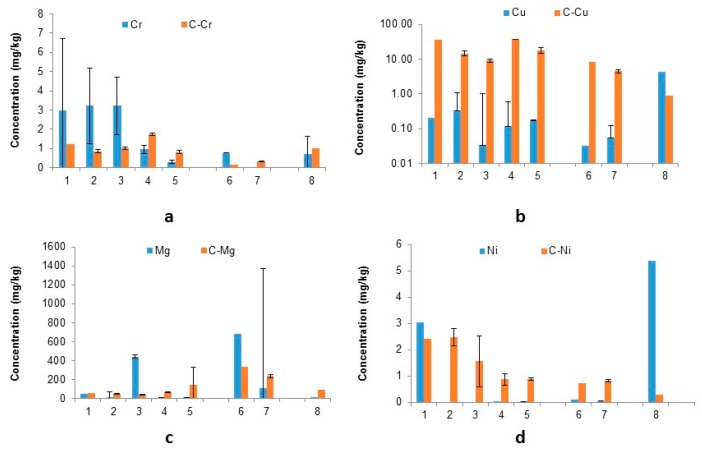
Concentration of (**a**) Cr, (**b**) Cu, (**c**) Mg and (**d**) Ni (mg/kg) in solution after water and citric acid leach (C-Element refers to citrate leached results). (Samples: ESP dust: 1–5, BF sludge: 6–7, BOF sludge: 8)

The elements Cr, Cu, Mg and Ni are often reported as components of steel waste and are known problematic PTEs environmentally [10,11,61,62] and therefore included in this study (Figure 4).

The liberation of chromium is very specific to the type of waste being treated; BOF fine sludge shows that the treatment of citrate successfully increases the amount of chromium leached out into solution (40%). As the oxidation state of Cr is considered a key-contributing factor to its solubility its liberation may possibly be attributed to the sample composition. Trivalent chromium compounds, with the exception of acetate, hexahydrate of chloride, and nitrate salts, are generally insoluble in water. Zinc and lead salts of chromic acid are practically insoluble in cold water and alkaline metal salts (e.g., calcium, strontium) of chromic acid are slightly soluble in water [66] Copper shows the same pattern for ESP dust and BF sludge (Figure 4) with a significant increase in Cu concentration (Table 3) in leachate after citrate treatment (4356–32,000% increase).

The coarser samples (sinter ESP dust and BF sludge) show a higher concentration of chromium in the water-leached samples than citrate-leached (58–73% and 80% decrease respectively). The reasons for this apparent paradoxical behaviour are not yet well understood, Different cation exchanges taking place where the Na-triCitrate solution may interact, via adsorptions, mechanical entrapments or co-precipitation. This will ultimately result in chemisorption, physisorption, electrostatic interactions and/or dipole-dipole interactions resulting in complexes that result in metals such as Cr to form a new non-soluble complex for example onto CaCO_3_ sludge [67]. For samples 4 and 5, like in the case of calcium, do not follow the ESP dust trend, but show a relative increase in the release of Cr when contacted with citrate solution (83–176%).

Concentrations of magnesium (Figure 4) with the exception of samples 3 and 6 (50–91% decrease), all show a significant increase in magnesium concentrations (Table 3) after citrate leaching (15–1300% increase). With ten replicates it is difficult to determine whether these outlying samples are anomalies or a result in lack of homogeneity, which is a recognised issue with studies of these wastes [64]. The trend for nickel (Figure 4) in BOF fine sludge shows a decrease (94.89%) of Ni released with citrate treatment relative to water leaching; this showed successful immobilisation by citrate. All other sample types (excluding 1) show greatly improved liberation of Ni with citrate treatment (665–1.15 × 10^4^%).

### 3.2. Results: Stabilisation with BF Slag

The stabilisation of steel wastes was explored using BF slag as a stabilisation agent. Moulds were mixed and set (over a 28-day period) at different ratios of steel waste by-product to BF slag (50/50, 30/70 and 70/30), and subjected to the BS EN 12457-3 WAC leaching test, where the respective eluents were totalled (mg/kg) and used for comparison to assess the potential of BF slag as a stabiliser. This data was used instead of WAC values for ease of comparison.

As a procedural note if values are quoted as 100+ (positive or negative) this shows values exceeding the total leached values from initial characterisation. This is to emphasise the variation in data from replicate samples compared to the original measurements, which is a known issue for this material [64]. Elements shown in these results are the measurable (quantifiable) concentrations in both the untreated and stabilised waste and that other PTEs were found to be below the LOD/inert classification with normal WAC testing.

#### 3.2.1. Sinter ESP Dust

**Figure 5:**
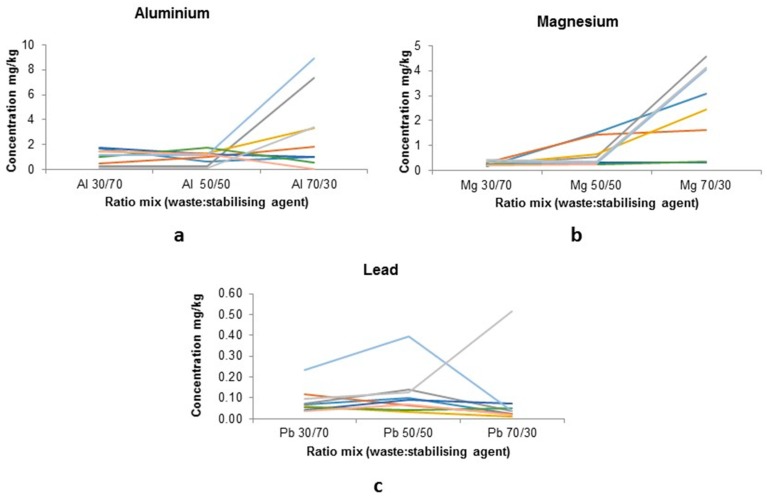
Total PTE content in leachate of sinter ESP dust and BF slag mix: (**a**) Al; (**b**) Mg; and (**c**) Pb—different colours represent nine different samples from the sinter plant.

Sinter ESP dust: slag moulds leachate concentrations are shown for PTEs Al, Mg and Pb in Figure 5, these PTEs were again chosen as a result of them showing the highest leachate concentrations. Various ESP samples were collected from different points and dates to show variability in production and labelled 1–9. The majority of other PTEs being investigated were below the LOD and are indicative of stabilisation occurring.

The mixture of sinter ESP dusts with BF slag (Figure 5) show a variation across the different mix ratios, where Al and Mg seem to follow a similar pattern with the 70/30 mix shows the highest leachate concentration, whilst the Pb content does not show much variation.

There is a large increase in Mg and Al leachate concentrations for the 70/30 (waste:slag) mix, with over 100%+ increase. The concentrations measured in the 30/70 mix are also higher than the 50/50 value by 60–100%. The level of Al in the 70/30 mixes compared to the 50/50, for four samples (1, 5, 6, and 8, decrease ~41% with the latter reduced by 99%). This variation may be a result of the specific locations of the ESP dust retrieval, i.e., 5 and 6 were identified as coming from the hopper that has yet to be altered by the sintering process (Figure 1).

Levels of lead also vary: 50/50 (0.031–0.396 mg/kg), 30/70 (0.036–0.232 mg/kg) and 70/30 (0.009–0.518 mg/kg), with average results 50/50: 0.118 mg/kg, 30/70: 0.087 mg/kg and 70/30: 0.089 mg/kg, suggesting that the 50/50 mix has the highest available Pb concentration (by 26%).

These preliminary results make it difficult to conclusively pick the best ratio therefore results were compared to data from total waste materials leachate, to determine the difference.

**Figure 6:**
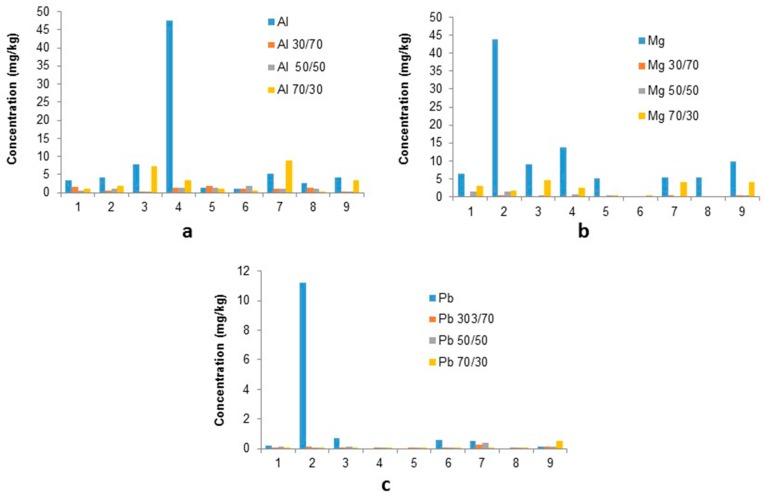
leachate concentrations in stabilised moulds compared to raw sinter ESP dusts: (**a**) Al; (**b**) Mg; and (**c**) Pb.

Figure 6 shows for all three of the PTEs (Al, Mg and Pb), the majority of samples show the leachate from 100% ESP dust to be significantly higher than stabilised mixtures, which confirms the success of stabilisation. There are 3 primary ESP samples (taken on different days of plant production) with similar concentrations for Al which exceed the 100% waste materials leachate in the 70/30 mixes. E.g., sample 3: has a 70/30 leachate concentration of 7.32 ± 0.20 mg/kg and the raw sample produced 7.9 ± 1 mg/kg, or sample 7 has a leachate concentration of 8.94 ± 0.30 mg/kg from the 70/30 mix, compared to the raw samples 5.2 ± 1.8mg/kg (71% increase). Mg levels from the 70/30 mix are lower than the raw sample leachate concentrations: 23–96% lower, with only two samples having less than a 50% reduction (3 and 7). A reduction in Pb levels has also been observed by 87–100%.

All stabilised moulds for BF slag with ESP dusts, showed a significant reduction in leachability, however in order to utilise our waste by-products numerical evaluations were carried on the 50/50 and 70/30 mixes (Table 4 and Table 5) in order to determine the best ratio for the reduction of key PTE in leachate.

The majority of elements monitored decrease significantly in leachate; 80–100% in the 50/50 stabilised moulds compared to the 100% ESP dusts (Table 4), indicating successful stabilisation. The Pb level identified for sample 9 (−27.3%) was treated as an outlier for this sample probably due to sample heterogeneity.

The 70/30 mixes show a lower reduction for constituents Al, Ca, Fe and Mg (Table 5), and the results for K are erratic, even when reductions are observed the values varies greatly 18–100%. The key PTEs Pb and Zn however both show a similar/slightly improved reduction with the higher waste content; the median level of lead rose from 80% to 89% and zinc remained between 98% and 99%.

#### 3.2.2. Blast Furnace Sludge

**Figure 7:**
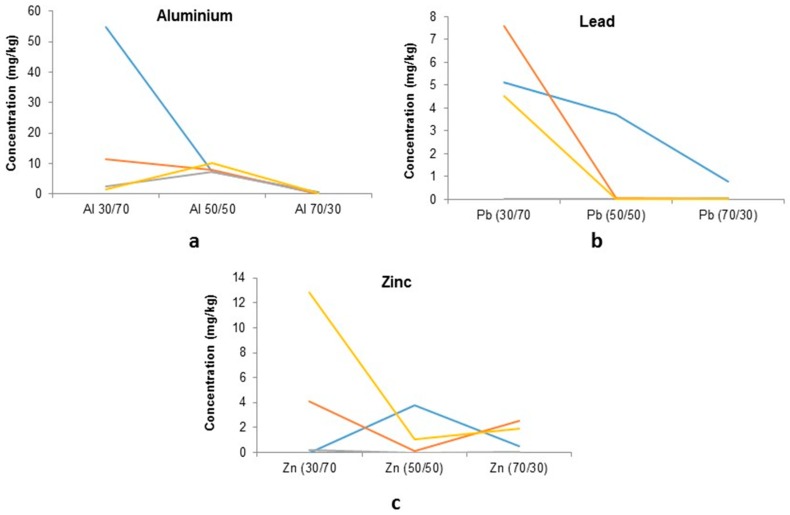
Total leached PTE concentrations from stabilised BF sludge and BF Slag: (**a**) Al; (**b**) Pb; and (**c**) Zn—different colours represent four different samples from the blast furnace.

For the majority of BF sludge samples, PTEs were not detected suggesting that stabilisation has taken place; Pb, Zn and Al were all measurable in leachates values are shown in Figure 7.

There is a clear trend observed where the stabilising agent (BF slag) influences the amount of Pb leached from the various ratio mixes; the concentration from 70/30 mixes were 5.1, 7.6 and 4.5 mg/kg compared to the 30/70 mix; 0.78, 0.21 and 0.05 respectively. This is also seen in samples 2 + 4 for Al with leachate concentrations of 54.9 and 7.5 to 7.9 mg/kg and undetectable by the 70/30 mix.

**Figure 8:**
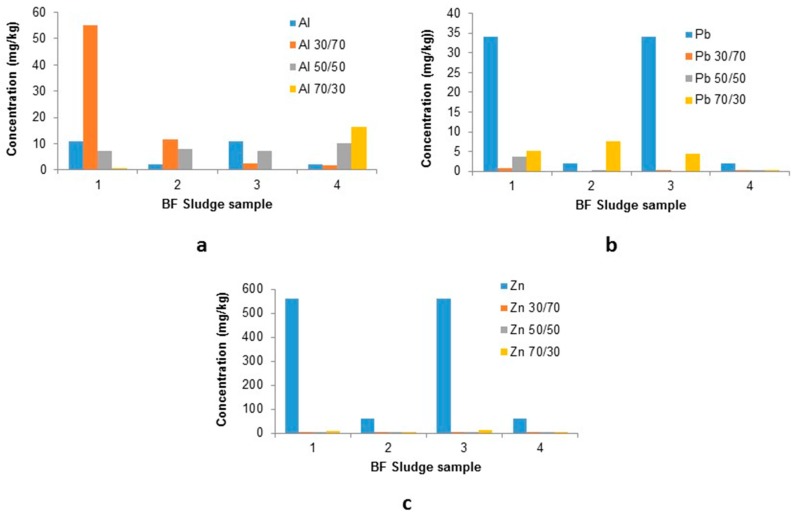
leachate concentrations in stabilised moulds compared to raw sinter ESP dusts: (**a**) Al; (**b**) Pb; and (**c**) Zn.

A similar trend where the BF slag influences leachate concentrations of zinc can be observed; a higher concentration is present in the 2nd sample, with 4 mg/kg (30/70 mix) and 2.5 mg/kg (70/30 mix). The 4th sample has 12.8 mg/kg in the 30/70 leachate and only 1.9mg/kg leached from the 70/30 mix. The other two sub-samples (1 + 3) highlight how variable BF sludge can be, with opposite trends observed. This followed with a comparison of the raw BF sludge leachate with the mixed leachates to determine the success of the stabilisation process (Figure 8).

There is a clear reduction in the levels of lead and zinc compared to their raw leachate levels, with zinc stabilisation being more successful; e.g., sample 2, raw leachate level of 58.4 ± 30.7 mg/kg compared to the 70/30 mix—4.09 ± 0.15 mg/kg and sample 3, raw—559 ± 1,660 mg/kg to 7.4 ± 0.13 mg/kg at a 70/30 mix. The raw samples show a high variation in leached components whereas the 70/30 mix shows better reproducibility with its lower levels.

Aluminium levels show an increase in concentration for all stabilised mixes suggesting as with other samples Al is not as strongly sorbed as other components and therefore liberated into solution. This led to further investigation of both: 50/50 and 70/30 moulds, to evaluate the potential reduction for Al (Table 6 and Table 7).

As with the sinter ESP and FF dusts there is a clear reduction in PTE leachate concentrations for the 50/50 mix: Ca, Mg, and Zn in particular had 100% reduction (Table 6). Mn shows a similar reduction for 3 of the sample replicates (96–100+ reduction), however sample 1 shows one sample to have an increase in leachate concentration (85%). Levels of Fe were shown to increase for all samples (+100%). Lead levels vary between the replicates but still shows a reduction in leachate concentrations (42–100%).

The 70/30 BF sludge to BF slag mix (Table 7) shows high reduction levels again for Mg, Mn and Zn (98–100%), with lead levels showing a higher reduction (85–99%). Fe has completely changed and now shows a reduction in its leachate concentration (48–94%). As with the 50/50 results, there is a split outcome with regards to Al leachate concentrations i.e., sample 1 shows a 93% reduction whereas 2 increased by over 100%. Calcium and potassium levels still show a reduction but not as significant as the 50/50 mixes: Ca—43—100%.

These results suggest that for the key PTEs Pb and Zn show high reduction levels for both the 50/50 and 70/30 mixes, whereas these two mixes offer benefits for different PTEs and therefore provide options for better management in the industrial life cycle.

#### 3.2.3. BOF Fine Sludge

**Figure 9:**
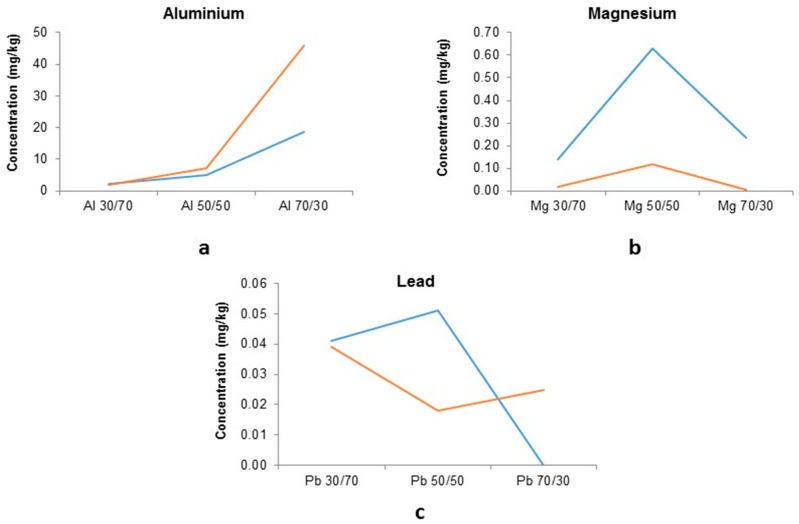
Leached PTEs from stabilised BOF fine sludge: Slag waste: (**a**) Al; (**b**) Mg; and (**c**) Pb different colours represent two different samples from the BOF.

BOF fine sludge samples were mixed with BF slag at ratios 30/70, 50/50 and 70/30, with PTEs Al, Mg and Pb showing detectable levels for all three mould ratios and therefore are displayed in Figure 9.

There is a high variation in leached content for the PTEs of interest (Figure 9): Aluminium shows the typical trend of increasing in leachate concentrations with higher BOF sludge content: 50/50: 5–7 mg/kg, 30/70: 1–2.5 mg/kg and 70/30: 18–45 mg/kg. Magnesium however shows its highest leachate seen in the 50/50 mix: 0.1–0.6 mg/kg.

The amount of Pb leached varies for the different replicates of the BOF fine sludge with the exception of the 30/70 (0.039–0.041 mg/kg), and the replicates have very different responses for the 50/50 and 70/30 mixes. One replicate shows an increase in the 70/30 mix of 38% (0.018–0.025 mg/kg) compared to its 50/50 mix which has the lowest measured leachate.

**Figure 10:**
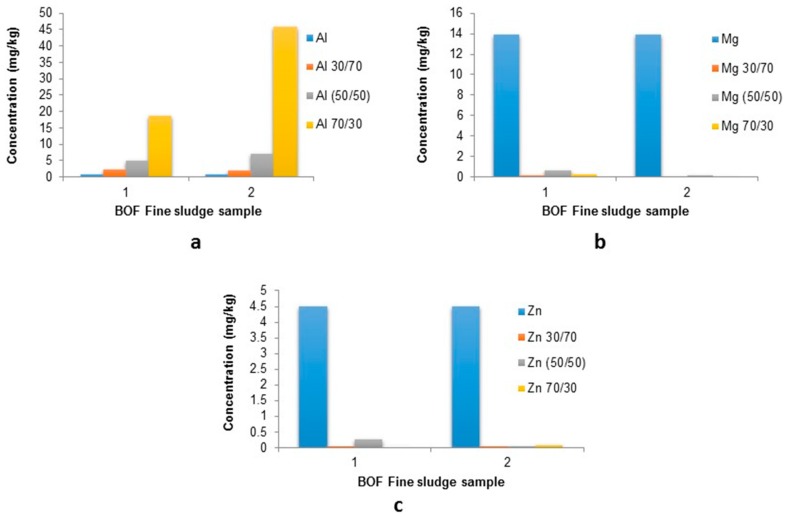
leachate concentrations in stabilized moulds compared to raw BOF fine sludge: (**a**) Al; (**b**) Mg; and (**c**) Zn.

No discrete trend in response is observed for the stabilisation, these results are compared to the total BOF fine sludge leachates (Figure 10).

The leachates produced from stabilisation experiments show a large reduction in Mg and Zn levels, e.g., Mg shows 13.9 ± 10.8 mg/kg leached from the total sample but only 0.631 ± 0.1 mg/kg from the 50/50 mixed mould. This is also the case of zinc with 4.5 ± 5.3 mg/kg from the total sludge and only 0.262 ± 0.33 mg/kg from the 50/50 mix. Aluminium levels show an increase in all stabilised moulds suggesting the BF slag is a likely source of available Al.

These results suggest that a 70/30 mix offers the highest potential for stabilisation of BOF fine sludge, however a 50/50 mix offers an optimum utilisation of both waste samples and therefore both mixes are investigated further (Table 8 and Table 9).

Calcium levels could not be validated due to lack of detection after stabilisation, which is also the case for lead levels, which suggests stabilisation in the 50/50 mix (Table 8). Iron levels cannot be validated by this method as measurable levels of iron in sample 2 suggests a reduction in leachate content, whereas sample 1 suggests that this process is results in higher mobilisation of iron.

Levels of aluminium also suggest that the mix of the BOF sludge and BF slag is quickly reaching Al saturation and therefore being desorbed into solution. Zn, Mg and Mn show a high reduction in their leaching (94–100%). The comparison with the 70/30 mixed leachate concentrations shows a similar reduction for Mn, Pb and Zn of 90–100+% whereas Mg levels show a less significant reduction: 66–99%.

It is worth noting that although only one replicate showed valid comparable data for both Ca and Fe they demonstrate positive results with 93–100% reduction in concentration. Finally, aluminium levels again increased in leachability substantially i.e., +100%.

## 4. Discussion

### 4.1. Citric Acid Stabilisation

The main elements of interest (K, Pb and Zn) show varying results from the leaching approach. These can be explained in part by the co-liberation or immobilisation of other elements e.g., Fe, Mn, Ca, Al, Cu, Ni and Cr. Lead has been successfully leached into solution from BF sludge, BOF sludge and ESP dusts using 0.1 M citrate solutions. This indicates that a lower concentration of Pb remained in the solid sample, resulting in a lower environmental threat. Similar results were obtained for zinc in BF sludge and BOF sludge. However, in sinter ESP dusts it appears that zinc is immobilised by the citrate solution due to ionic potential and exchange into a solid or non-soluble complex.

The liberation of potassium by 0.1 M-citrate solution was successful for BF sludge, but the results proved inconclusive for Sinter ESP dust due to the variability of results, which showed both immobilisation and liberation in solution. Leaching of K from BOF fine sludge was less effective in citrate than in water indicating that immobilisation of K occurs with citrate solution.

Iron and manganese were, as predicted, leached out in greater quantities with citrate solution than with water, probably due to dissolution of their associated hydroxides and strong soluble citrate complex formation. This may explain the enhanced concentrations of Pb, Zn and some other elements in citrate leachates; as such species can be desorbed from hydroxides at low pH. Additionally, as Fe and Mn ions compete to occupy available sorption sites.

For the effective potential application in industrial processing, it is important to assess whether the addition of a citrate solution has simply resulted in solubilisation of PTE into the leachate or has it successfully significantly reduced the availability of these elements in the residual solid waste, and ultimately reduced its hazard potential. The Waste Acceptance Criteria (WAC) testing indicated that the bulk BF, BOF and ESP dusts were classified as “inert” therefore making standard regulatory reference levels inappropriate for a reactive material. Consequently, we evaluated the effectiveness of the citrate treatments against the percentage difference of the pre and post-treated waste materials to demonstrate the potential this technique to reduce toxicity hazard. Data are presented in Figure 5, which shows the percentage decrease of the key waste constituents (K, Pb and Zn) relative to the original total elemental concentrations.

**Figure 11:**
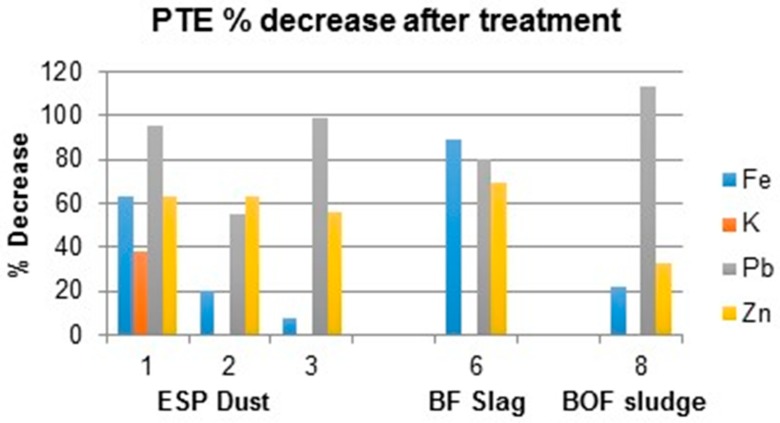
Percentage decrease of PTE concentration (mg/kg) after citrate treatment.

From Figure 11 it can be seen there is a variation in the percentage decrease (or increase) of total PTE concentrations in the residual waste samples (for clarity error bars are removed). Additionally, where K measurements are absent, values are < analytical detection limit after treatment.

Lead and zinc both follow similar trends and show a decrease in residual solid concentrations: Pb (median of 88% ± 20.2) and Zn (61% decrease ± 12.8). The large decrease of Pb (113%) suggests total liberation in BOF fine sludge, however as previously identified [68], the total content varies in concentration and the median value does not account for fluctuations, which could explain the higher percentage. Potassium however shows an increase in blast furnace sludge (78%) that suggests, as might be anticipated that water was a good leaching agent for K, whereas the citrate is causing a complexing effect holding K in solid complexes. There is no detectable difference in K levels for some samples whereby further analysis would be required for validation. As water washing has been proven an efficient method for potassium removal [69] meaning citrate may have no conclusive impact.

### 4.2. BF Stabilisation

There is no definitive trend that can be observed within the different waste types and the elemental concentrations in the varying waste:slag mixes, that can be applied to all of the PTEs measured. The majority of samples show their lowest PTE concentrations to be from the 50/50 mixed, ‘stabilised’ moulds, which were either similar or slightly higher in concentration than the 30:70 mixes. Further comparison of the leached concentrations with their respective raw samples was carried out, which demonstrated that the majority of samples including the 70/30 showed significant reduction levels between 90–100%.

They did however tend to show an increase in Al concentration, for both the 50/50 and 70/30 mixed ratios suggesting release from the stabilising agent. It does seem that optimal use of a 50/50 mixture of the two waste types will create a stabilised material that might be suitable for landfill.

Additionally, it is worth considering that as with other stabilisation materials, e.g., Portland cement, the products may be appropriate for other industrial/commercial uses. Initial tests of larger mould for compressive strength and potential use in construction were found to be too weak after a 28-day curing period.

## 5. Conclusions

The different approaches evaluated in order to test stabilisation or immobilisation of a number of steel process by-products resulted in a range of outcomes. These are compelling results which demonstrate for the first time potential for active risk reduction of wastes which represent enormous environmental burden worldwide.

The experimental results from stabilisation by mixing the wastes with Portland cements shows that a mixture of 70% waste material and 30% PC offers a 90%+ reduction in leachate concentrations compared to the raw materials themselves. This means that although the key PTEs cannot be recovered for re-use, landfilling with an inert classification would be possible. The same outcome can be observed when using BF slag as a stabilising agent; with the exception of aluminium a significant reduction can be seen with the 50/50 waste:stabiliser mixes with a reduction of over 90%. This may provide direct economic advantage as diversion from hazardous classification and premium landfill tax, once additional processing costs are considered.

The application of citric acid demonstrated an ability to substantially reduce or stabilise PTEs within the various steel production by-products (dust, sludge). For our key PTEs we see a 73% decrease in release from sinter dust i.e., stabilised within its matrix, as opposed to being liberated (78%) from the of BF sludge. Lead and zinc however both showed significant leaching into solution after treatment with citrate solution (averaging 50–80% and 50–60% decrease in total solid concentration). This results in process solids that are greatly reduced in elements of concern (Pb and Zn) and the iron rich solid wastes could be either recycled back into the blast furnace or landfilled as inert wastes.

Both applications show the potential of two treatment approaches in which waste materials can be reclassified from hazardous/non-hazardous to inert. A variety of secondary applications are feasible such as direct recycling back into plant, production of a bulk construction material or even to harbour valuable metals e.g., where Pb and Zn markets are relatively buoyant for additional income. Ultimately treatment allows for better waste management through reduction in negative impacts environmentally and reduction in long term risk for human health.

## Figures and Tables

**Table 1:** Percentage increase* in key constituents K, Pb and Zn, after citrate leaching.

		% Increase with Citrate Solution		
	Sample	K	Pb	Zn
**Sinter ESP dust**	1	0	114	−96
	2	75	3 × 10^5^	−98
	3	−8	948	−39
	4	0	8612	−31
	5	2 × 10^8^	1007	0
**BF Sludge**	6	90	5110	186
	7	2 × 10^8^	4470	2380
**BOF fine sludge**	8	−36	1080	175

*Negative values denote a reduction in potentially toxic elements (PTE) concentration found in leachate compared to total waste.

**Table 2:** Percentage increase* in key constituents; Al, Ca, Fe and Mn, after citrate leaching.

		% Increase with Citrate Solution			
	Sample	Al	Ca	Fe	Mn
**Sinter ESP dust**	1	1270	15	3 × 10^5^	40
	2	328	−52	14,330	282
	3	29,270	−75	2 × 10^8^	93
	4	5860		2 × 10^8^	3 × 10^5^
	5	4020		2 × 10^8^	
**BF Sludge**	6	4268	77	2746	2250
	7	16,280	66	24,790	383
**BOF fine sludge**	8	4677	−3	49,540	6600

*Negative values denote a reduction in PTE concentration found in leachate compared to total waste.

**Table 3:** Percentage increase* of key constituents after citrate leaching.

	% Increase with Citrate Solution				
	Sample	Cr	Cu	Mg	Ni
**Sinter ESP dust**	1	−59	17,440	37	−21
	2	−74	4360	232	2 × 10^7^
	3	−69	3 × 10^4^	−92	2 × 10^7^
	4	84	3 × 10^4^	1100	3460
	5	176	1 × 10^4^	1365	11,525
**BF Sludge**	6	−81	26,069	−51	665
	7	0	8280	118	1696
**BOF fine sludge**	8	40	−79	537	−95

*Negative values denote a reduction in PTE concentration found in leachate.

**Table 4:** Percentage reduction of PTEs from 50/50 stabilised moulds compared to raw Sinter ESP dust.

ID	Al	Ca	Fe	Mg	Mn	Pb	Zn
**Mean**	90.0	93.4	103.7	93.7	92.5	60.7	95.7
**STDEV**	11.6	13.2	1.5	6.7	15.0	46.5	5.2

**Table 5:** Percentage reduction of PTEs from 70/30 stabilised moulds compared to raw Sinter ESP dust.

ID	Al	Ca	Fe	Mg	Mn	Pb	Zn
**Mean**	54.1	89.3	80.9	81.1	100.0	6.3	95.1
**STDEV**	31.6	12.1	28.2	21.9	0.5	207.8	6.1

**Table 6:** Percentage reduction of PTEs from 50/50 stabilised moulds compared to raw BF Sludge.

ID	Al	Ca	Fe	Mg	Mn	Pb	Zn
**1**	32.8	100.0	−100+	100.0	−85.2	88.9	100.0
**2**	92.9	100.0	−100+	100.0	96.1	99.7	100.0
**3**	−266.7	100.0	−100+	99.9	100+	42.5	100.0
**4**	−416.3	100.0	−100+	100.0	99.6	98.6	100.0

**Table 7:** Percentage reduction of PTEs from 70/30 stabilised moulds compared to raw BF Sludge.

ID	Al	Ca	Fe	Mg	Mn	Pb	Zn
**1**	93.5	100.0	97.3	100.0	100.0	85.0	98.7
**3**	−725.4	43.4	47.7	97.7	99.8	98.9	99.7

**Table 8:** Percentage reduction of PTEs from 50/50 stabilised moulds compared to raw BOF fine Sludge.

ID	Al	Ca	Fe	Mg	Mn	Pb	Zn
**1**	−615.4		−2498.7	95.5	96.9		94.2
**2**	−916.8		101.6	99.2	100.0		99.7

**Table 9:** Percentage reduction of PTEs from 70/30 stabilised moulds compared to raw BOF fine Sludge.

ID	Al	Ca	Fe	Mg	Mn	Pb	Zn
**1**	−100		92.9	66.3	100.0	102.4	100.4
**2**	−100	101.3		99.5	98.7	96.4	90.9
